# Complement-related molecular classification and a gene signature for lung adenocarcinoma

**DOI:** 10.1186/s40164-023-00388-0

**Published:** 2023-02-21

**Authors:** Lin Zhang, Yannan Yang, Weihao Lin, Fei Shao, Yibo Gao, Jie He

**Affiliations:** 1 Department of Thoracic Surgery, National Cancer Center/National Clinical Research Center for Cancer/Cancer Hospital, Chinese Academy of Medical Sciences and Peking Union Medical College, Beijing, China; 2 State Key Laboratory of Molecular Oncology, National Cancer Center/National Clinical Research Center for Cancer/Cancer Hospital, Chinese Academy of Medical Sciences and Peking Union Medical College, Beijing, China; 3 Department of Oncology, Renmin Hospital of Wuhan University, Wuhan, China; 4 Laboratory of Translational Medicine, National Cancer Center/National Clinical Research Center for Cancer/Cancer Hospital, Chinese Academy of Medical Sciences and Peking Union Medical College, Beijing, China; 5 Central Laboratory & Shenzhen Key Laboratory of Epigenetics and Precision Medicine for Cancers, National Cancer Center/National Clinical Research Center for Cancer/Cancer Hospital & Shenzhen Hospital, Chinese Academy of Medical Sciences and Peking Union Medical College, Shenzhen, China; 6 State Key Laboratory of Molecular Oncology, National Cancer Center/National Clinical Research Center for Cancer/Cancer Hospital, Chinese Academy of Medical Sciences and Peking Union Medical College, Beijing, China

**Keywords:** Lung adenocarcinoma, Gene signature, Complement, TCGA, GEO

## Abstract

**Background:**

Lung adenocarcinoma (LUAD) is a major cause of cancer-related death worldwide, and the roles of complement-related genes in it have not been thoroughly investigated yet. In the study, we aimed to systemically examine the prognostic performance of complement-related genes, classify the patients into two different clusters and stratify the patients into different risk groups using a complement-related gene signature.

**Methods:**

To achieve this, clustering analyses, Kaplan–Meier survival analyses, immune infiltration analyses were performed. LUAD patients from The Cancer Genome Atlas (TCGA) were classified into two subtypes (C1 and C2). A prognostic signature, consisting of four complement-related genes, was established using TCGA-LUAD cohort and validated in six Gene Expression Omnibus datasets and an independent cohort from our center.

**Results:**

The prognosis of C2 patients is better than that of C1 patients and the prognosis of low risk patients is significantly better than high risk patients across the public datasets. In our cohort, the OS of patients in low risk group is better than that in high risk group but the difference is not significant. Patients with a lower risk score were characterized by a higher immune score, a higher level of BTLA, higher infiltration levels of T cells, B lineage, myeloid dendritic cells, neutrophils, endothelial cells, and a lower infiltration level of fibroblast.

**Conclusions:**

In summary, our study has established a new classification method and developed a prognostic signature for LUAD, while future studies are needed for further exploration of the underlying mechanism.

**Supplementary Information:**

The online version contains supplementary material available at 10.1186/s40164-023-00388-0.

To the editor,

Lung cancer remains the leading cause of cancer-related mortality (18.0% of the total cancer deaths), with non-small cell lung cancer (NSCLC) as the main pathological type, accounting for approximately 80–85% of cases [1]. The complement system is a critical component of innate immunity, and is tightly regulated and activated by three distinct pathways: the classical pathway, via antigen–antibody complexes; the alternative pathway, via any surface that is not specifically protected by complement regulators; and the lectin pathway, via binding of pattern-recognizing mannose-binding lectins to carbohydrate ligands on the surface of pathogens [2]. Since complement system plays dual roles in tumor development and previous studies focused on limited complement proteins, comprehensive analyses of complement-related genes and tumor microenvironment (TME) in clinical cohorts are needed.

In this study, the list of complement-related genes was obtained from the AmiGO 2 Web portal (http://amigo.geneontology.org/amigo/landing) and was further supplemented by genes gathered from published articles [3–5]. We integrated the transcriptomic data of lung adenocarcinoma (LUAD) from The Cancer Genome Atlas (TCGA) and identified two distinct complement clusters according to indexes such as cophenetic and dispersion, and there is a significant difference in overall survival between clusters (Fig. 1A–D). Moreover, we divided the patients from TCGA-LUAD into a training cohort and a test cohort at a ratio of 1:1, and constructed a robust complement-related gene signature composed of 4 genes (C1QBP, C1QTNF6, C1QTNF9 and CR2) using the training cohort (Fig. 1F). The risk score of the signature was calculated as follows: risk score = (0.3377 × EXP_*C1QBP*_) + (0.4692 × EXP_*C1QTNF6*_)—(1.4672 × EXP_*C1QTNF9*_)—(0.2512 × EXP_*CR2*_). The clinical information of the patients, complement gene expression patterns, as well as the correspondence between cluster, risk, and survival were displayed (Fig. 1E, G). The prognostic efficacy of the signature was then validated in test cohort and whole cohort of TCGA-LUAD (Fig. 1H–P). The signature showed satisfying performance in difference clinical subgroups such as stage, age, and gender (Fig. 1Q–S). Subsequently, the signature was validated in six Gene Expression Omnibus (GEO) cohorts (GSE13213, GSE19188, GSE30219, GSE31210, GSE41271, GSE50081) and could effectively distinguish patients with different OS in most situations (Fig. 2A–F). For further validation, we collected LUAD samples from Cancer Hospital, Chinese Academy of Medical Sciences (CHCAMS) and performed immunohistochemistry. The information of the patients was shown in Additional file 1. The signature could distinguish patients with different survival outcomes in this cohort although the difference was not significant (Fig. 2G). The effects of individual genes were shown in Fig. 2H–I. In TCGA-LUAD (Fig. 2H), all four genes could distinguish patients’ survival outcomes effectively, while in CHCAMS cohort, only the result of C1QTNF6 was significant (Fig. 2I). Representative immunohistochemistry images were shown in Additional file 2: Fig. S5J. To explore potential underlying mechanisms, we first evaluated the infiltrated immune cells using CIBERSORT that contained the LM22 algorithm [6] and revealed that the infiltration levels of T cells, B lineage cells, myeloid dendritic cells, neutrophils and endothelial cells were significantly higher in the low risk group compared to the high risk group in TCGA-LUAD (Fig. 2J). We then evaluated the TME using the ESTIMATE algorithm [7] and revealed that immune score was significantly higher in low risk group (Fig. 2K).

**Fig. 1:**
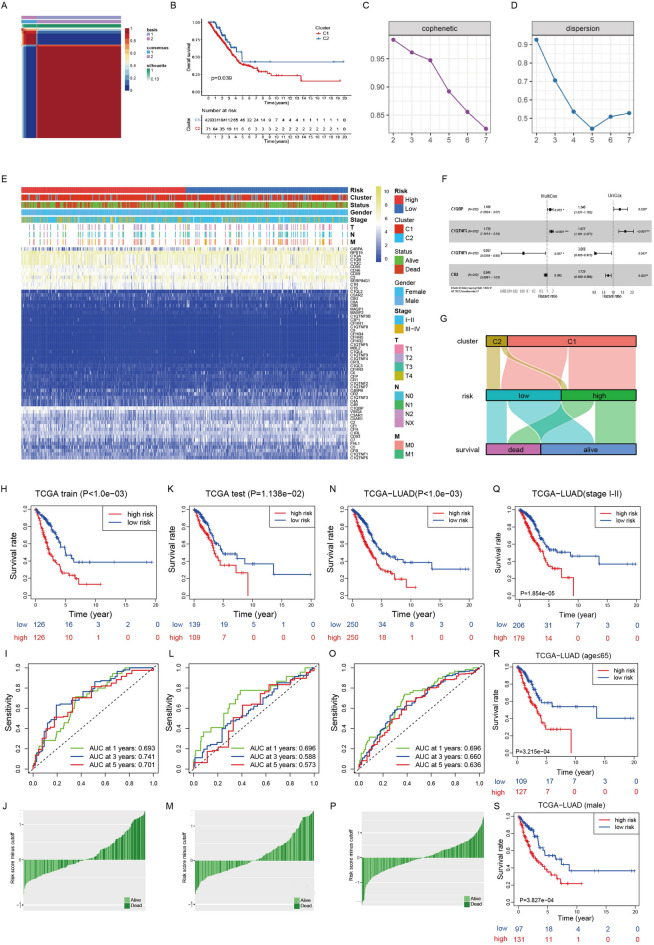
Construction of the complement-related gene signature and internal validation using TCGA-LUAD. **A** Heatmap of NMF clustering for complement-related genes in TCGA-LUAD cohort with two clusters. **B** Kaplan–Meier curves of overall survival in TCGA-LUAD on the basis of the complement-related clusters. **C–D** The relationships between cophenetic, dispersion and the numbers of clusters. **E** Heatmap showing the expression patterns of 64 complement-related genes between different risk groups, and clusters in the TCGA-LUAD cohort. Survival status, gender, age and TNM stage were the annotations. **F** Forrest plot showing the four genes in the signature. **G** Alluvial diagram showing the relationship between cluster, risk, and survival. Kaplan–Meier curves of overall survival between the low and high risk groups based on the median risk score were performed in training set **H**, test set **K** and whole set **N** of TCGA-LUAD. ROCs of the signature for prediction of overall survival at 1, 3 and 5 years in training set **I**, test set **L** and whole set **O** of TCGA-LUAD. Distribution of the risk scores of patients in training set **J**, test set **M** and whole set **P** of TCGA-LUAD. Kaplan–Meier curves of overall survival between the low and high risk groups in stage I-II **Q**, age ≤ 65 **R**, and male **S** subgroups of TCGA-LUAD cohort. TCGA-LUAD, lung adenocarcinoma cohort from The Cancer Genome Atlas; ROC, receiver operating characteristic curve

**Fig. 2:**
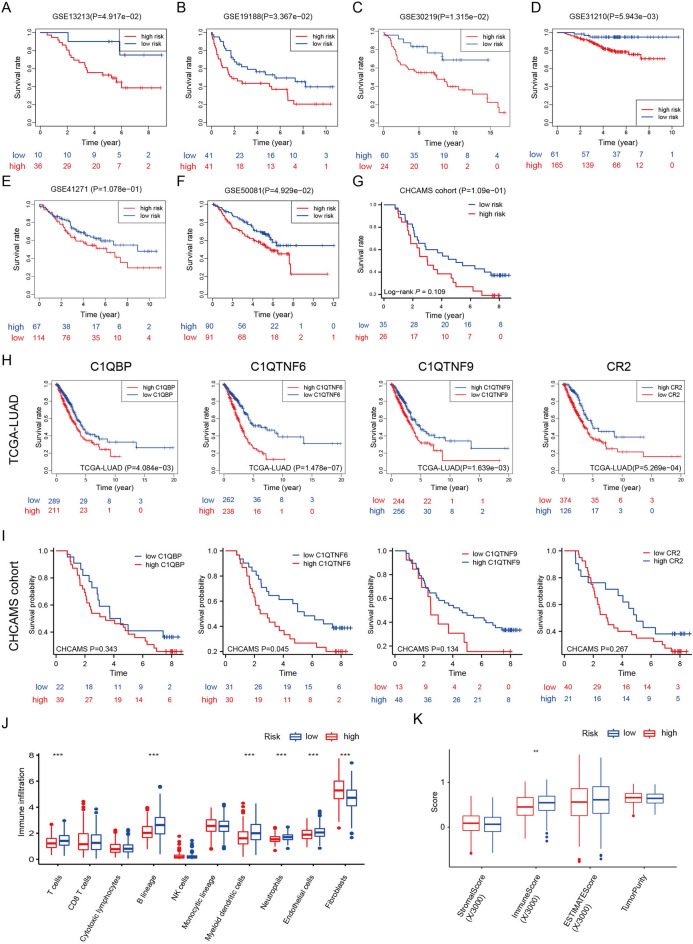
External validation of the complement-related gene signature. **A**–**G** Kaplan–Meier curves of overall survival between the low and high risk groups based on the median risk scores or the optimal cut off values in GSE13213, GSE19188, GSE30219, GSE31210, GSE41271, GSE50081, and CHCAMS cohorts. **H** Prognostic effects of each signature gene in TCGA-LUAD. **I** Prognostic effects of each signature gene in CHCAMS cohort. **J** Assessment of the abundance of infiltrated immune cells between the low and high risk groups in TCGA-LUAD. ^***^P < 0.001. **K** Comparison of Estimation of STromal and Immune Cells in MAlignant Tumours using Expression Data scores between low and high risk groups in TCGA-LUAD. ^**^P < 0.01. TCGA-LUAD, lung adenocarcinoma cohort from The Cancer Genome Atlas; CHCAMS, Cancer Hospital, Chinese Academy of Medical Sciences

To our knowledge, there has been no study that identified LUAD subtypes according to complement-related genes and constructed a gene signature based on these genes for prediction of prognoses of LUAD. The signature has not only been tested internally in TCGA-LUAD, but also validated in six GEO datasets and an independent cohort from our center. Hopefully, it may be used as a tool to identify high risk LUAD patients a for individualized therapies.

In conclusion, the complement-related gene signature may serve as a prognostic biomarker for LUAD patients and future studies on this may help to improve its validity.

## Supplementary Information

**Additional file 1: Table S1.** CHCAMS cohort information.

**Additional file 2.** The complete content of this study.

## Data Availability

The datasets used in the current study are available from the corresponding author upon reasonable request.
