# Supplementary material for: Complement-related molecular classification and a gene signature for lung adenocarcinoma
Source: Exp Hematol Oncol. 2023 Feb 21;12:22. doi: 10.1186/s40164-023-00388-0 (PMC9942347; doi:10.1186/s40164-023-00388-0)
Supplement: Supplementary file 2 — Additional file 2. The complete content of this study. [file 40164_2023_388_MOESM2_ESM.docx]

For the better understanding of our study, we provided a full-text article that incorporate complete analysis result and detailed methods.

**Complement-related molecular classification and a gene signature for lung adenocarcinoma**

**Running title**: Complement-related clustering and signature for LUAD

Lin Zhang^1,2,3†^ Yannan Yang^1,2†^ Weihao Lin^1,2^ Fei Shao^1,2,4^ Yibo Gao^5,1,2,4^ Jie He^1,2,4^

^1^ Department of Thoracic Surgery, National Cancer Center/National Clinical Research Center for Cancer/Cancer Hospital, Chinese Academy of Medical Sciences and Peking Union Medical College, Beijing, China.

^2^ State Key Laboratory of Molecular Oncology, National Cancer Center/National Clinical Research Center for Cancer/Cancer Hospital, Chinese Academy of Medical Sciences and Peking Union Medical College, Beijing, China.

^3^ Department of Oncology, Renmin Hospital of Wuhan University, Wuhan, China.

^4^ Laboratory of Translational Medicine, National Cancer Center/National Clinical Research Center for Cancer/Cancer Hospital, Chinese Academy of Medical Sciences and Peking Union Medical College, Beijing, China.

^5^ Central Laboratory & Shenzhen Key Laboratory of Epigenetics and Precision Medicine for Cancers, National Cancer Center/National Clinical Research Center for Cancer/Cancer Hospital & Shenzhen Hospital, Chinese Academy of Medical Sciences and Peking Union Medical College, Shenzhen, China.

^†^These authors have contributed equally to this work and share first authorship

*Correspondence:

Yibo Gao [gaoyibo@cicams.ac.cn](mailto:lichunxiang@cicams.ac.cn) Tel: +86-010-67781331

Jie He [hejie@cicams.ac.cn](mailto:Heprof.jiehe@gmail.com) Tel: +86-010-87788798

**ABSTRACT**

**Background**: Lung adenocarcinoma (LUAD) is a major cause of cancer-related death worldwide, and the roles of complement-related genes in it have not been thoroughly investigated yet. In the study, we aimed to systemically examine the prognostic performance of complement-related genes, classify the patients into two different clusters and stratify the prognosis of LUAD patients into different risk groups using a complement-related gene signature.

**Methods**: To achieve this, clustering analyses, Kaplan-Meier survival analyses, pathway enrichment analyses, immune infiltration analyses were performed. LUAD patients from The Cancer Genome Atlas (TCGA) were classified into two subtypes (C1 and C2). A prognostic signature, consisting of four complement-related genes, was established using TCGA-LUAD cohort and validated in six Gene Expression Omnibus datasets.

**Results**: The prognosis of C2 patients is better than that of C1 patients and the prognosis of low risk patients is significantly better than high risk patients across the datasets. Patients with a lower risk score were characterized by a higher immune score, a higher level of BTLA, higher infiltration levels of T cells, B lineage, myeloid dendritic cells, neutrophils, endothelial cells, and a lower infiltration level of fibroblast. In enrichment analyses, pathways such as complement activation alternative pathway, ether lipid metabolism, are enriched in low risk group, while those such as cell cycle, DNA replication are enriched in high risk group.

**Conclusions**: In summary, our study has established a new classification method and developed a prognostic signature for LUAD, while future studies are needed for further exploration of the underlying mechanism.

**Key words** lung adenocarcinoma; gene signature; complement; TCGA; GEO

**INTRODUCTION**

Lung cancer remains the leading cause of cancer-related mortality (18.0% of the total cancer deaths), with non-small cell lung cancer (NSCLC) being the main pathological type, accounting for approximately 80-85% of cases[1]. Lung adenocarcinoma (LUAD) is the most common type of NSCLC and has a high mortality rate [2]. In the recent decade, immune checkpoint inhibitors (ICIs), especially anti-PD-1/PD-L1 therapy, have revolutionized the landscape of cancer treatment due to the intermediate response rates to a wide variety of cancer types [3]. However, despite the great success brought by immunotherapy, especially anti-PD-1/PD-L1 therapy, moderate response rate, acquired resistance to immunotherapy and finally inevitable progression of NSCLC raise challenges to such treatment[4]. Thus, a deeper insight into the immune system and tumor microenvironment (TME) is urgently needed to identify the patients who are at high risk or are likely to respond to immunotherapy.

The complement system is a critical component of the innate immune system and the TME, as its cascade generates membrane attack complexes (C5b-9) on the surface of invading pathogens or target cells through activation of anaphylatoxins C3 and C5[5]. The complement system is tightly regulated and activated by three distinct pathways: the classical pathway (CP), via antigen-antibody complexes; the alternative pathway (AP), via any surface that is not specifically protected by complement regulators; and the lectin pathway (LP), via binding of pattern-recognizing mannose-binding lectins to carbohydrate ligands on the surface of pathogens[6]. Of note, in addition to being the first line defense against invasive pathogens, the complement system also participates in adaptive immune response, embryogenesis and homeostasis[7].

Due to the complexity of the complement proteins, the potential role of complement system in specific TME is undetermined. With innate cytotoxic effects on antibody-binding malignant cells, complement inhibits tumor development through formation of membrane attack complexes[8]. However, complement can be protumoral through promoting immunosuppression and chronic inflammation in TME. For example, high levels of complement regulators in TME, which is capable of suppressing complement activation, protects tumor cells from the attack of complement and contributes to the tumor immune escape mechanisms[9]. Moreover, C5a has been found to exert protumorigenic effect through dampening anti-tumor immune mediated by modulation of regulatory T cell and production of immunosuppressive cytokines[10].

Since complement system plays dual roles in tumor development and previous studies focused on limited complement proteins, comprehensive analyses of complement-related genes and TME in clinical cohorts are needed. In this study, we integrated the transcriptomic data from The Cancer Genome Atlas (TCGA) and identified two distinct complement clusters, among which significant survival differences, diverse clinical characteristic, and different infiltrated immune cells were observed. Moreover, we constructed a robust complement-related prognosis (CRP) model composed of 4 genes (C1QBP, C1QTNF6, C1QTNF9 and CR2), prognostic efficacy of which was subsequently validated by 6 Gene Expression Omnibus (GEO) cohorts (GSE13213, GSE19188, GSE30219, GSE31210, GSE41271, GSE50081). Notably, patients in high-risk CRP group may potentially benefit from immunotherapy due to the significantly upregulated expression of CD274. In conclusion, our results revealed that complement plays critical roles in LUAD and that the CRP model may be a potential predictive biomarker for prognosis and immunotherapy response.

**METHODS**

*Lung adenocarcinoma datasets acquisition and preprocessing*

Public lung adenocarcinoma (LUAD) transcriptome datasets were obtained from TCGA and GEO. A total of seven datasets (TCGA-LUAD, GSE13213, GSE19188, GSE30219, GSE31210, GSE41271, GSE50081) with both corresponding transcriptomic data and clinical information were acquired for analysis. Entrez IDs in the transcriptomic data were transformed into official gene symbols based on a GTF file downloaded from GENCODE (<https://www.gencodegenes.org/>). The patients in TCGA-LUAD were randomly assigned to a training set and a test set by a ratio of 1:1.

*Clustering analyses based on complement-related genes*

The list of complement-related genes was obtained from the AmiGO 2 Web portal (<http://amigo.geneontology.org/amigo/landing>), further supplemented by genes gathered from published articles[11-13] and confirmed using the Gene database (https://www.ncbi.nlm.nih.gov/gene/?term=). A non-negative matrix factorization (NMF)[14] clustering algorithm was performed through R package ‘NMF’ to identify distinct complement related clusters based on the expression profiles of 60 complement-related genes. The optimal clustering number was determined based on the cophenetic, dispersion and silhouette coefficients.

*Immune infiltration analysis and prediction of immunotherapy efficacy*

The proportion of infiltrated immune cells in the tumor microenvironment was estimated by CIBERSORT algorithm[15], a computational approach reinforced by support vector regression to quantify 22 immune cell fractions. To further infer the potential benefit of immunotherapy for each patient, the expression levels of several prominent checkpoints were extracted from the expression matrix. Moreover, Estimation of STromal and Immune Cells in MAlignant Tumours using Expression Data (ESTIMATE) score was assessed through R package ‘estimate’ to reveal the fraction of immune cells and stromal cells in tumor [16].

*Construction of complement-related prognostic model*

After performing univariate Cox regression for each gene in TCGA-LUAD cohort, 7 unique genes with a *P* value < 0.05 were identified as prognosis-associated genes. To construct a CRP signature, the least absolute shrinkage and selection operator (LASSO) method was applied through R package ‘glmnet’ based on the expression level of the prognosis-related genes[17]. Moreover, a stepwise Cox proportional hazards regression model was used to optimize the model, in which one standard error above the minimum criteria was selected. After the multivariate Cox analysis of the complement-related genes, a risk score formula was calculated by considering both the expression of optimized genes and estimated Cox regression coefficients: risk score = (Gene1 expression × Gene1 coefficient) + (Gene2 expression × Gene2 coefficient)+ (Gene3 expression × Gene3 coefficient)+ (Gene4 expression × Gene4 coefficient). After calculating the risk score of each sample in the TCGA-LUAD training cohort, samples were stratified into low-risk and high-risk groups based on the median risk score. The performance of the CRP signature was evaluated by the Kaplan-Meier survival curve and the time-dependent receiver operating characteristic (ROC) curves.

*Validation of the CRP signature in multiple validation cohorts*

For internal validation of the prognostic value of CRP signature, the test set and complete TCGA-LUAD cohort were used. The risk score was first calculated for each sample based on the formula derived from the training cohort. Then, samples were divided into low-risk and high-risk groups according to the median risk score determined in the TCGA-LUAD training cohort. For external validation, patients in GSE13213, GSE19188, GSE30219, GSE31210, GSE41271 and GSE50081 were stratified based on the median risk score or the optimal cutoff values.

*Biological pathways enrichment analysis*

Gene Set Enrichment Analysis (GSEA, <http://www.gsea-msigdb.org/>) and Gene Set Variation Analysis (GSVA) were conducted[18]. The enrichment scores of each sample were calculated according to the annotated gene sets c5.go.v7.3.symbols.gmt and c2.cp.kegg.v7.3.symbols.gmt. The number of permutations was set at 1000 and gene sizes smaller than 15 or larger than 500 were excluded. A gene set with normalized *P* value < 0.05 and adjusted *P* value < 0.05 was considered an enriched group.

*Clinical specimens collection*

61 surgically resected, formalin-fixed, paraffin-embedded LUAD tissues were retrospectively collected from the biobank of Cancer Hospital, Chinese Academy of Medical Sciences (CHCAMS). Waiver of informed consent was obtained from the Ethics Committee of CHCAMS, in consideration of the retrospective nature of the study. The clinicopathological data of the enrolled patients in CHCAMS cohort were shown in **Supplementary Table 1**.

*Immunohistochemistry (IHC) staining and evaluation*

The tissue microarray slide was stained with primary antibodies against human C1QBP (dilution 1:1000; CST, #6502), C1QTNF6 (dilution 1:100; Thermo, PA5-51674), C1QTNF9 (dilution 1:200; Sigma, HPA056632), CR2 (dilution 1:20; Proteintech, 24374-1-AP). The immunohistochemical staining was evaluated using an H-score, which consists of the percentage of positive cells (0=1-10%, 1=11-40%, 2=41-70%, 3=71-100%) and the staining intensity (0=very weak, 1 = weak, 2 = moderate, 3 = strong)[19]. All immunostained tissues were blindly reviewed by two pathologists (Z.C. and X.F.).

*Statistical analysis*

Data analyses were conducted on R version 4.0.5 and GraphPad Prism 8.0.2. Univariate and multivariate Cox regression analyses alone with hazard ratios and 95% confidence intervals were performed through R package ‘survival’ (https://cran.r-project.org/package=survival). The differences of various clinical factors were analyzed by independent *t* test. A *P* value < 0.05 was considered statistically significant.

**RESULTS**

***Identification of distinct complement-related patterns in LUAD patients***

The flow chart of this study is shown in **Figure 1**. To develop the complement-related expression patterns in LUAD, the expression profiles of complement-related genes were combined with the clinical information of TCGA-LUAD patients. Further, we stratified patients into two distinct clusters based on the expression profiles of the genes using an NMF algorithm (429 cases in Cluster 1 (C1), 71 cases in Cluster 2 (C2), **Supplementary Table 2**, **Fig. S1A, B**). The Kaplan-Meier survival analysis showed that patients in C2 had a better prognosis than those in C1 (*P* = 0.039 in overall survival, **Fig. 2A**; *P* = 0.307 in progression free survival, **Fig. 2B**). The clinical information and gene expression patterns were displayed in **Fig. 2C**. With a better prognosis, patients in C2 were predominantly enriched in early clinical stages and an M0 metastasis status, which may explain the prognostic difference between clusters to some extent. However, when further assessing the potential efficacy of immunotherapy between clusters, evaluation of infiltrated immune cells and immune checkpoint-related genes was performed and the results revealed little differences between clusters (**Fig. 2D, E**).

***Construction of a complement-related prognostic signature in TCGA-LUAD training cohort***

To construct a CRP signature, univariate Cox regression analysis was performed in TCGA-LUAD cohort with corresponding clinical information and 7 genes were prognostically significant (*P* < 0.05, **Supplementary Table 3**). LASSO analysis was then performed and lambda.min was applied to minimize overfitting in TCGA-LUAD training cohort (**Fig. S2A, B**). A complement-related prognostic signature was constructed and included four genes: C1QBP, C1QTNF6, C1QTNF9, CR2. Next, multivariate Cox analysis was performed to build a stepwise Cox proportional hazards regression model with the four genes mentioned above (**Fig. S2C**). The risk score of the CRP signature was calculated as follows: risk score = (0.3377 × EXP*_C1QBP_*) + (0.4692 × EXP*_C1QTNF6_*) – (1.4672 × EXP*_C1QTNF9_*) - (0.2512 × EXP*_CR2_*) (**Supplementary Table 4**).

***Evaluation and validation of the predictive performance of the complement-related prognostic signature***

To evaluate the prognostic efficacy of the constructed signature, cases in the training cohort of TCGA were stratified into high and low risk score groups according to the median of total risk score, and the distribution of risk scores, survival time and expression profiles of signature genes of each patient was shown in **Fig. 3G**. Then the Kaplan-Meier survival analysis was performed and showed that patients with high risk score had a significantly worse prognosis (*P* < 0.0001, **Fig. 3A**). In addition, areas under the curve (AUC) of the CRP signature at 1, 3 and 5 years were 0.693, 0.741 and 0.701, respectively (**Fig. 3D**).

Then, TCGA-LUAD test cohort and complete TCGA-LUAD cohort were used and stratified based on the median risk score as mentioned above (**Fig. 3H**-**I**), respectively. Significant worse overall survival (OS) was observed in the patients with a high risk score (**Fig. 3B, C**) and the AUCs at 1, 3 and 5 years were 0.696, 0.588, 0.573 (**Fig. 3E**) and 0.696, 0.660, 0.636 (**Fig. 3F**), respectively. In addition, CRP discriminated high risk patients with worse prognoses in different subgroups with different clinicopathological features, including TNM stage (**Fig. 4G, H**), age (**Fig. 4I, J**) and gender (**Fig. 4K, L**). Of note, survival of patients with different risks was different in those with an early TNM stage, while the difference was not significant in those with an advanced stage (**Fig.4G,H**). So we explored the distribution of the risk score in patients of different stages (**Fig. S4H, I**). Notably, the difference was significant between patients with an early stage and a late stage (**Fig.S4H**), but was not significant between stage II and III, or stage III and IV (**Fig.S4I**).

For external validation, patients from six GEO cohorts (GSE13213, GSE19188, GSE31210, GSE30219, GSE41217, GSE50081) with corresponding clinical information were enrolled and stratified based on their median risk scores or optimal cut off values. Consistent and significant differences were observed in GSE13213 (*P* < 0.05, **Fig. 4A**), GSE19188 (*P* < 0.05, **Fig. 4B**), GSE31210 (*P* < 0.01, **Fig. 4C**), GSE30219 (*P* < 0.05, **Fig. 4D**), GSE50081 (*P* < 0.05, **Fig. 4F**), but not in GSE41271 (*P*＞0.05, **Fig. 4E**). And most of the AUCs at 1, 3, and 5 years for the cohorts were acceptable (**Fig. S4A-F**).

***Identification and validation of the signature in an independent cohort***

In order to further validate the signature, we used an independent cohort from CHCAMS that involved 61 LUAD patients. We performed immunohistochemistry and calculated the H-scores. The effects of the four signature genes on OS in TCGA-LUAD were all significant (**Fig. 5A-D**). However, in CHCAMS cohort, only C1QTNF6 could distinguish patients’ OS with a significant result (**Fig. 5E-H**). When patients were divided into high or low risk groups according to the risk score, a difference on OS was observed although the difference was not significant (**Fig. 5I**). Representative images of the immunohistochemistry results were shown in **Fig. 5J**.

***Immune microenvironment and biological pathways enrichment assessment between different risk groups***

The association between complement and immune infiltration among multiple cancer types has been revealed and explored (5, 7). Thus, to further assess the immune microenvironment between different risk groups, we first evaluated the infiltrated immune cells using CIBERSORT that contained the LM22 algorithm (**Supplementary Table 5**). Enriched infiltration of T cells, B lineage cells, myeloid dendritic cells, neutrophils and endothelial cells were predominantly observed in the low risk group with better prognosis (**Fig. 6A**). Consistently, T cells, B lineage cells and myeloid dendritic cells were elevated in the patients of an early TNM stage (**Fig. S4J**). Subsequent evaluation of the TME using ESTIMATE revealed that immune score was significantly higher in low risk group (*P* < 0.01, **Fig. S5C, Supplementary Table 6**). Furthermore, correlation analyses between several prominent immunotherapeutic biomarkers and the CRP risk score were performed. The results showed that the expression level of BTLA was significantly higher in low risk group than that in high risk group (**Fig. S5D**). The results above implied that patients in high risk group may potentially benefit from immunotherapy.

To investigate the biological pathway underlying the distinct outcomes between patients from high or low CRP risk groups, biological pathways enrichment analyses using GSEA and GSVA were performed in TCGA-LUAD cohort. The top 10 significant GO terms and KEGG pathways positively and negatively correlated to CRP risk scores were shown in **Fig. 6B-E** and **Fig. S5A, B**. The high CRP risk group was significantly enriched in genes involved with DNA replication, homologous recombination and mismatch repair, while the low CRP risk group was markedly enriched in metabolism associated genes, including fatty acid metabolism, lipid metabolism, linoleic acid metabolism and retinol metabolism (**Fig. 6B-E, Fig. S5A, B**).

**DISCUSSION**

LUAD is a fatal malignancy in the world and remains to be the leading cause of cancer-related death[1]. The current situation has highlighted the urgent need to develop a signature to predict prognosis and response to immunotherapy. Complement is an important component of the innate immune system, and acts on several cell types in the TME[8]. The role of complement in tumor is context-dependent, it can both be promotive and suppressive[8]. In LUAD, the blockade of C5a results in a substantial improvement in the efficacy of anti-PD-1 antibodies against lung cancer growth and metastasis[20]. In the present study, we comprehensively analyzed the role of complement-related clusters in clinical implication and immune landscape in LUAD and built a gene signature for prediction of prognoses. Hopefully, this may contribute to personalized medicine for LUAD patients.

On the basis of the expression profiles of the complement-related genes, patients from TCGA-LUAD were classified into two clusters with distinct survival outcomes. We found that cluster 1 patients had a worse prognosis. However, while the difference for overall survival is significant, that for disease-free survival is not. Also, when performing immune infiltration analyses and immune checkpoint genes analyses, there are not many differences between clusters. The results have implicated that while the expressions of complement-related genes have an impact on patients’ survival, there may also be other important factors that should be included in clustering.

Then, a prognostic signature was developed using four complement-related genes, which could effectively stratify patients from TCGA-LUAD into high and low risk groups. Although showing a robust performance in public datasets, the signature was not perfectly validated in the immunohistochemistry assay in our cohort; considering that the complement system is intricate, there may be other important regulating factors in the translation process of the four genes. Conventionally, complement was considered to be initiated in three distinct pathways-classical, lectin and alternative[7]. C1q is the recognition molecule of complement classical pathway, among the four genes, C1QBP is a C1q-binding protein, with an aliase of gC1qR; C1QBP interact with globular target recognition domains of C1q, and is involved in the regulation of T cell immunity and cytokines[13]. C1QTNF6, with a full name of C1q and TNF related 6, is a member of C1QTNFs family, and is primarily expressed in tissues such as adipose tissues, liver, and lung[21]. Previous research has proved that the inhibition of C1QTNF6 could attenuate the proliferation, migration, invasion and promote apoptosis of NSCLC cells[22]; also, C1QTNF6 was upregulated in stage I LUAD tissues compared with adjacent non-cancerous tissues, C1QTNF6 knockdown could remarkably inhibit cell proliferation, migratory and invasive abilities, while overexpression of C1QTNF6 had an opposite effect[23]. CR2 is an aliase of CD21, and is a receptor for complement C3; expressed on the surface of B cells, CR2 allows the complement system to play a role in B cell activation and maturation[24]. It is revealed that an increased circulating level of CR2 is associated with better progression-free survival in patients with stage III NSCLC that receive concurrent chemoradiotherapy[25]. There have been no reports on C1QTNF9 in tumors yet. These genes may have indicated potential therapeutic strategies for LUAD treatment and further studies are still warranted to illustrate the functional roles of these four genes in the development of LUAD.

There has been many signatures on LUAD. For example, Liu J et al. have built a three-gene signature on pyroptosis-related lncRNAs that can accurately predict 1-, 3-, 5-year survival for TCGA-LUAD patients, it identified patients with a immune “hot” phenotype, and a high abundance of infiltrating immune cells that may implicate a higher sensitivity for PD1 and CTLA4 blockade [26]; Wang X et al. also constructed an m6A-related genes signature that could predict the prognoses of TCGA-LUAD patients, with a high degree of accuracy[27]. However, to our knowledge, this is the first study that identified LUAD subtypes according to complement-related genes and the first gene signature based on these genes for prediction of prognoses of LUAD. The signature has not only been tested internally in TCGA-LUAD, but also validated in six GEO datasets. It may be used as a tool to identify high risk LUAD patients a for individualized therapies.

Since the signature has shown good performance in different LUAD cohorts and clinical subgroups, we investigated the underlying possible mechanisms. Firstly, We analyzed the immune infiltration in the TME. The patients in low risk group have higher infiltration levels of T cells, B lineage, myeloid dendritic cells, neutrophils, endothelial cells, and a lower infiltration level of fibroblast. Interestingly, cancer-associated fibroblasts are the main cellular components of the tumor stroma, and can facilitate cancer cell migration and invasion[28]. Therefore, the infiltration of these cells may contribute to an anti-tumor TME in low risk patients and lower their mortality. Consistent with this, GSEA showed that GO/KEGG terms such as complement activation alternative pathway, ether lipid metabolism, are enriched in low risk group, while those such as cell cycle, DNA replication, DNA replication preinitiation complex are enriched in high risk group. In GSVA, a similar result was observed. The results implicated that that tumor cells in high risk group are in an active state of proliferation and that patients with a low risk score may have orchestrated the above-mentioned pathways to fight against tumors and reduce their risk of fatality.

There are a few limitations. First of all, this is a retrospective study that included mostly public cohorts. Secondly, the prediction for immunotherapy response was estimated indirectly because of the lack of an immunotherapy cohort. Thirdly, the conclusions need to be validated in future studies with large sample sizes.

In conclusion, we have clustered the patients in TCGA-LUAD into two different classes based on the expression profiles of identified complement-related genes. In addition, we constructed a complement-related gene signature that can predict overall survival and possible immunotherapy response of LUAD patients. The signature was validated in six cohorts from another public database and a cohort from our center, and could serve as a clinical tool for prediction. Future studies on this may help to improve its validity.

**Abbreviations**

AUC, areas under the curve; AP, the alternative pathway; CHCAMS, Cancer Hospital, Chinese Academy of Medical Sciences; CRP, complement-related prognosis; CP, the classical pathway; ESTIMATE, Estimation of STromal and Immune Cells in MAlignant Tumours using Expression Data; GEO, Gene Expression Omnibus; GSVA, Gene Set Variation Analysis; ICIs, immune checkpoint inhibitors; IHC, Immunohistochemistry; LP, the lectin pathway; LASSO, least absolute shrinkage and selection operator; LUAD, lung adenocarcinoma; NMF, non-negative matrix factorization, NSCLC, non-small cell lung cancer; OS, overall survival; ROC, receiver operating characteristic curve; TCGA, The Cancer Genome Atlas; TME, tumor microenvironment.

**Data availability**

The datasets used in the current study are available from the corresponding author upon reasonable request.

**Declarations**

**Ethics approval and consent to participate**

Ethical approval was obtained from the Ethics Committee of Cancer Hospital, Chinese Academy of Medical Sciences. Waiver of informed consent was obtained from the same committee in consideration of the retrospective nature of the study.

**Consent for publication**

Not applicable.

**Authors' contributions**

Z,L and Y,YN wrote the main manuscript text and performed bioinformatic analyses, L,WH and S,F were responsible for plotting figures and collecting samples, G,Yb and H,J were responsible for the revision of the manuscript and the supervision of the study. All authors read and approved the final manuscript.

**Funding**

This work was supported by the National Key R&D Program of China (2019YFC1315700), National Natural Science Foundation of China (82122053, 82188102), the Beijing Municipal Science & Technology Commission (Z191100006619115), R&D Program of Beijing Municipal Education Commission (KJZD20191002302), CAMS Initiative for Innovative Medicine (2021-1-I2M-012, 2021-1-I2M-015), Non-profit Central Research Institute Fund of Chinese Academy of Medical Sciences (2021-PT310-001), and Aiyou Foundation (KY201701).

**Competing interests**

The authors declare that there are no conflicts of interest.

**References:**

1. Siegel RL, Miller KD, Fuchs HE, Jemal A. Cancer Statistics, 2021. CA Cancer J Clin (2021) **71**: 7-33.

2. Sung H, Ferlay J, Siegel RL, Laversanne M, Soerjomataram I, Jemal A *et al*. Global Cancer Statistics 2020: GLOBOCAN Estimates of Incidence and Mortality Worldwide for 36 Cancers in 185 Countries. CA Cancer J Clin (2021) **71**: 209-249.

3. Ribas A, Wolchok JD. Cancer immunotherapy using checkpoint blockade. Science (2018) **359**: 1350-1355.

4. Draghi A, Chamberlain CA, Furness A, Donia M. Acquired resistance to cancer immunotherapy. Semin Immunopathol (2019) **41**: 31-40.

5. Afshar-Kharghan V. The role of the complement system in cancer. J Clin Invest (2017) **127**: 780-789.

6. Holers VM. Complement and its receptors: new insights into human disease. Annu Rev Immunol (2014) **32**: 433-459.

7. Roumenina LT, Daugan MV, Petitprez F, Sautes-Fridman C, Fridman WH. Context-dependent roles of complement in cancer. Nat Rev Cancer (2019) **19**: 698-715.

8. Reis ES, Mastellos DC, Ricklin D, Mantovani A, Lambris JD. Complement in cancer: untangling an intricate relationship. NAT REV IMMUNOL (2018) **18**: 5-18.

9. Beatty GL, Gladney WL. Immune escape mechanisms as a guide for cancer immunotherapy. Clin Cancer Res (2015) **21**: 687-692.

10. Ajona D, Ortiz-Espinosa S, Pio R. Complement anaphylatoxins C3a and C5a: Emerging roles in cancer progression and treatment. Semin Cell Dev Biol (2019) **85**: 153-163.

11. Merle NS, Church SE, Fremeaux-Bacchi V, Roumenina LT. Complement System Part I - Molecular Mechanisms of Activation and Regulation. FRONT IMMUNOL (2015) **6**: 262.

12. Merle NS, Noe R, Halbwachs-Mecarelli L, Fremeaux-Bacchi V, Roumenina LT. Complement System Part II: Role in Immunity. Front Immunol (2015) **6**: 257.

13. Ricklin D, Hajishengallis G, Yang K, Lambris JD. Complement: a key system for immune surveillance and homeostasis. Nat Immunol (2010) **11**: 785-797.

14. Gaujoux R, Seoighe C. A flexible R package for nonnegative matrix factorization. Bmc Bioinformatics (2010) **11**: 367.

15. Newman AM, Liu CL, Green MR, Gentles AJ, Feng W, Xu Y *et al*. Robust enumeration of cell subsets from tissue expression profiles. Nat Methods (2015) **12**: 453-457.

16. Yoshihara K, Shahmoradgoli M, Martinez E, Vegesna R, Kim H, Torres-Garcia W *et al*. Inferring tumour purity and stromal and immune cell admixture from expression data. Nat Commun (2013) **4**: 2612.

17. Friedman J, Hastie T, Tibshirani R. Regularization Paths for Generalized Linear Models via Coordinate Descent. J Stat Softw (2010) **33**: 1-22.

18. Ritchie ME, Phipson B, Wu D, Hu Y, Law CW, Shi W *et al*. limma powers differential expression analyses for RNA-sequencing and microarray studies. Nucleic Acids Res (2015) **43**: e47.

19. Wang W, Shao F, Yang X, Wang J, Zhu R, Yang Y *et al*. METTL3 promotes tumour development by decreasing APC expression mediated by APC mRNA N(6)-methyladenosine-dependent YTHDF binding. Nat Commun (2021) **12**: 3803.

20. Ajona D, Ortiz-Espinosa S, Moreno H, Lozano T, Pajares MJ, Agorreta J *et al*. A Combined PD-1/C5a Blockade Synergistically Protects against Lung Cancer Growth and Metastasis. Cancer Discov (2017) **7**: 694-703.

21. Schaffler A, Buechler C. CTRP family: linking immunity to metabolism. Trends Endocrinol Metab (2012) **23**: 194-204.

22. Zhang W, Feng G. C1QTNF6 regulates cell proliferation and apoptosis of NSCLC in vitro and in vivo. Biosci Rep (2021) **41**.

23. Lin G, Lin L, Lin H, Xu Y, Chen W, Liu Y *et al*. C1QTNF6 regulated by miR-29a-3p promotes proliferation and migration in stage I lung adenocarcinoma. Bmc Pulm Med (2022) **22**: 285.

24. Dunkelberger JR, Song WC. Complement and its role in innate and adaptive immune responses. Cell Res (2010) **20**: 34-50.

25. Vaes R, Reynders K, Sprooten J, Nevola KT, Rouschop K, Vooijs M *et al*. Identification of Potential Prognostic and Predictive Immunological Biomarkers in Patients with Stage I and Stage III Non-Small Cell Lung Cancer (NSCLC): A Prospective Exploratory Study. Cancers (Basel) (2021) **13**.

26. Liu J, Liu Q, Shen H, Liu Y, Wang Y, Wang G *et al*. Identification and Validation of a Three Pyroptosis-Related lncRNA Signature for Prognosis Prediction in Lung Adenocarcinoma. Front Genet (2022) **13**: 838624.

27. Wang X, Zhao C, Huang D, Liu Z, Liu M, Lin F *et al*. A Novel M6A-Related Genes Signature Can Impact the Immune Status and Predict the Prognosis and Drug Sensitivity of Lung Adenocarcinoma. Front Immunol (2022) **13**: 923533.

28. Kim D, Kim JS, Cheon I, Kim SR, Chun SH, Kim JJ *et al*. Identification and Characterization of Cancer-Associated Fibroblast Subpopulations in Lung Adenocarcinoma. Cancers (Basel) (2022) **14**.

**Figure legends**

**Figure 1 Flowchart of the present study.**


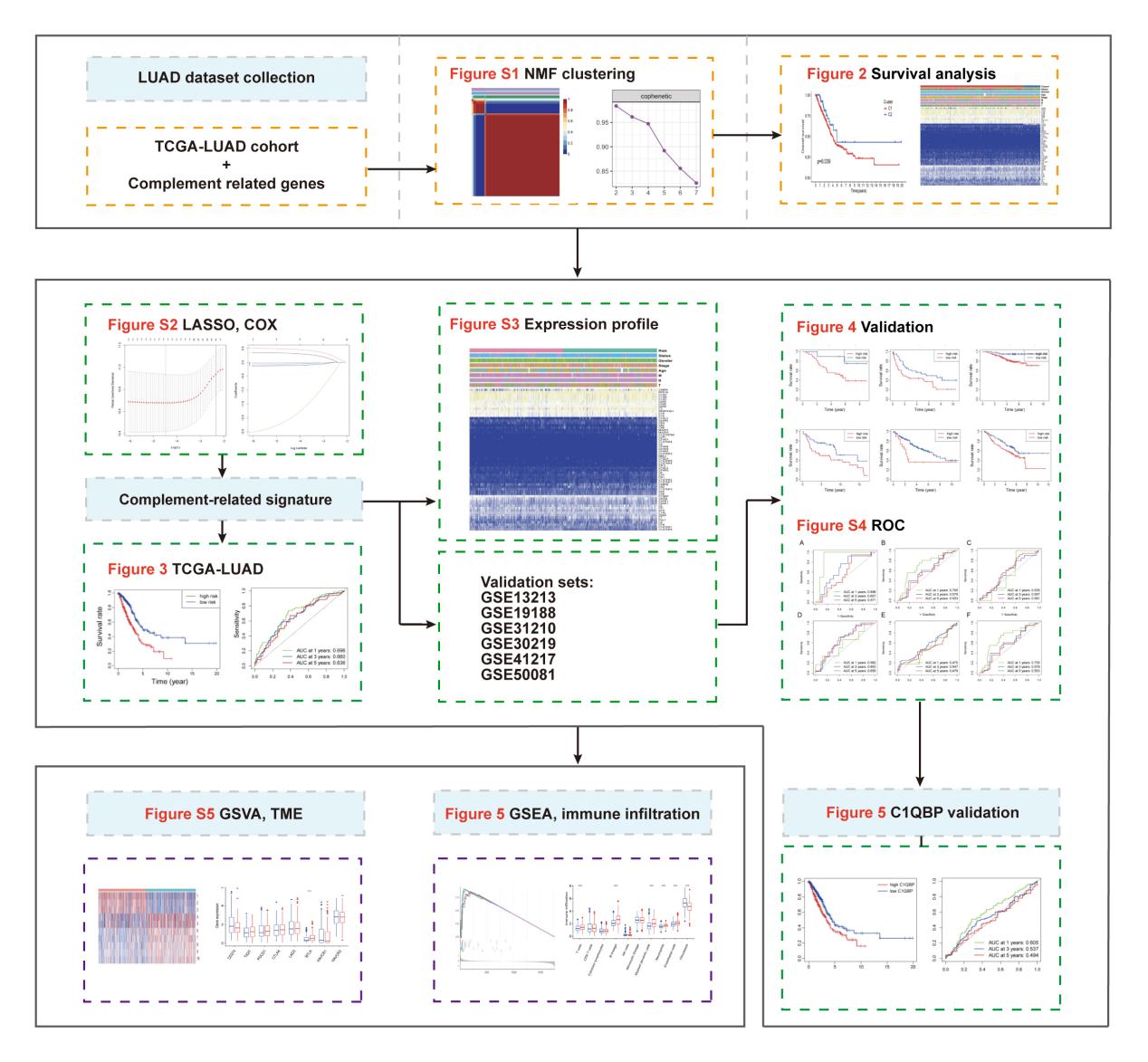


**Figure 2 Unsupervised clustering of complement-related genes and characteristics of complement-related clusters.**

**(A-B)** Kaplan-Meier curves of overall survival **(A)** and progression free survival **(B)** in **t**he TCGA-LUAD cohort on the basis of the complement-related clusters.

**(C)** Heatmap showing the expression patterns of the complement-related genes between cluster 1 and 2 in the TCGA-LUAD cohort. Survival status, gender, age and TNM stage were the annotations.

**(D- E)** Abundance of infiltrated immune cells **(D)** and differential expression of immune checkpoint-related genes **(E)** between the two complement-related clusters.


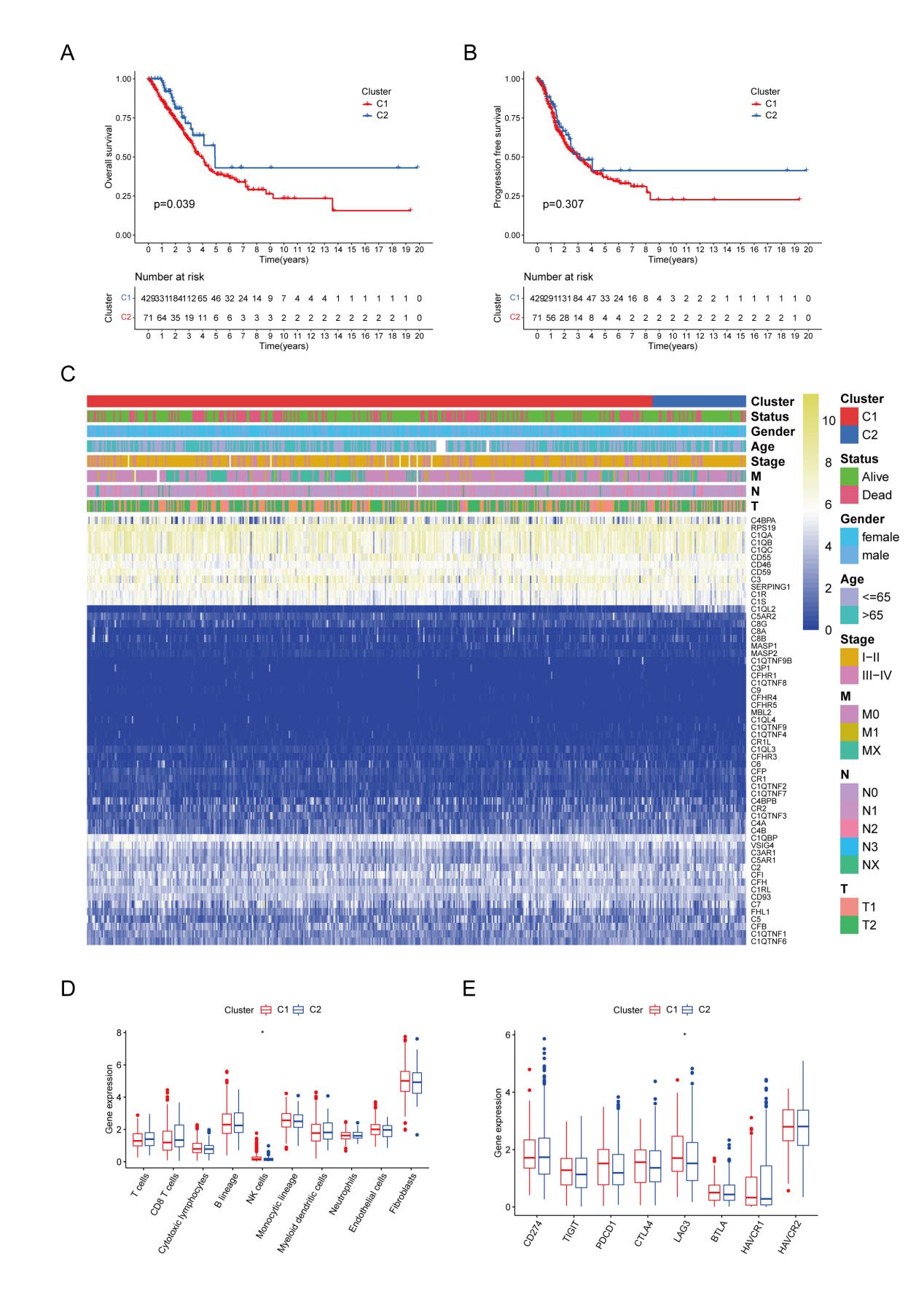


**Figure 3 Evaluation of the predictive performance of the complement-related signature.**

**(A-C)** Kaplan-Meier curves of overall survival between the low and high risk groups were performed in training set **(A)**, test set **(B)** and whole set **(C)** of TCGA-LUAD. Cutoff values were the median risk score of the training set (2.821420848).

**(D-F)** ROCs of the signature for prediction of overall survival at 1, 3 and 5 years in training set **(D)**, test set **(E)** and whole set **(F)** of TCGA-LUAD.

**(G-I)** The distribution of risk score, survival status and the expression level of four genes in training set **(G)**, test set **(H)** and whole set **(I)** of TCGA-LUAD.

**
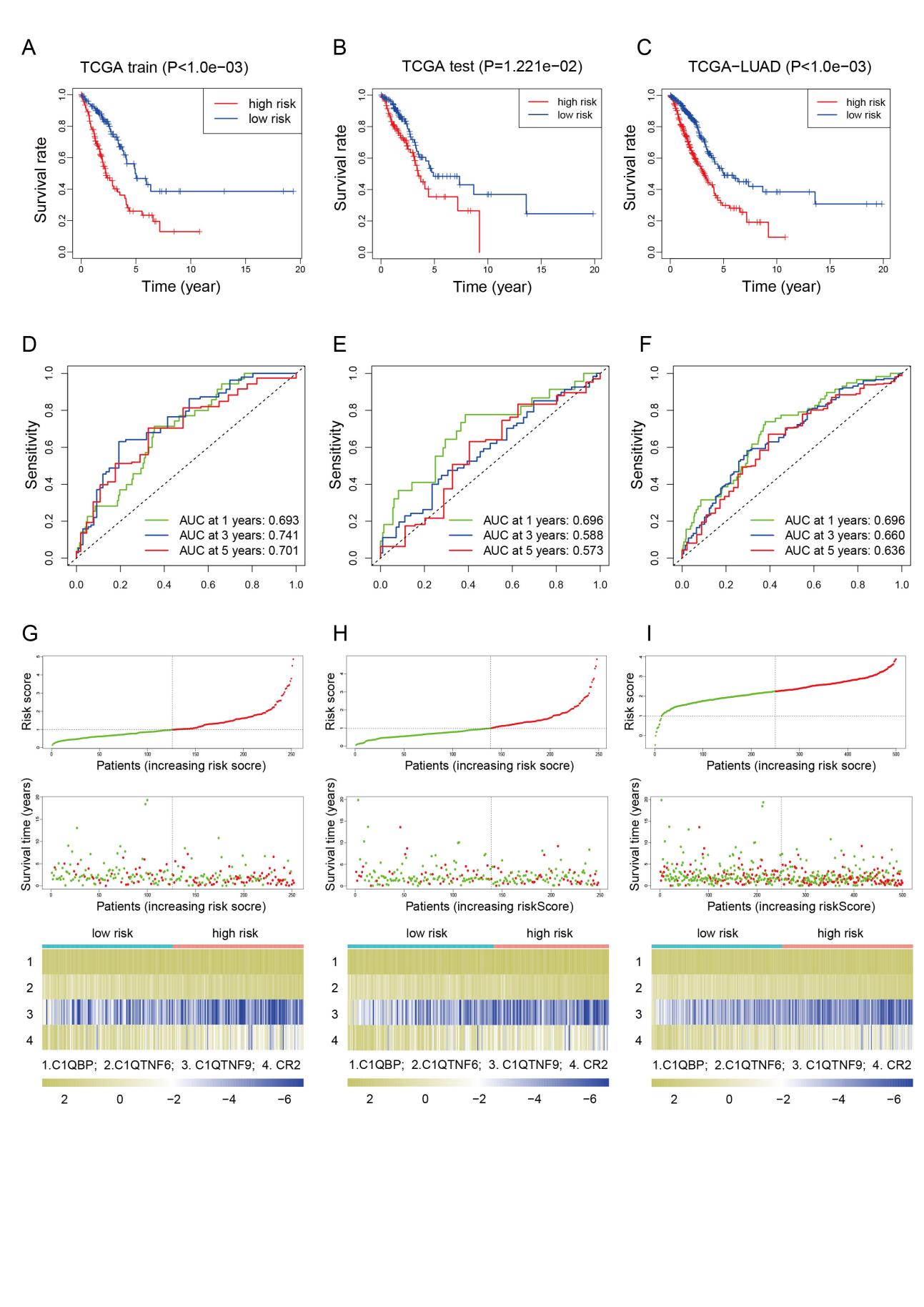
**

**Figure 4 Validation of the predictive performance of the complement-related signature.**

**(A-F)** Kaplan-Meier curves of overall survival between the low and high risk groups based on the median risk score or the optimal cut off values in GSE13213 (cutoff=-2.442324) **(A)**, GSE19188 (cutoff=0.157833209) **(B)**, GSE31210 (cutoff=-3.318957) **(C)**, GSE30219 (cutoff=-0.137467017) **(D)**, GSE41271 (cutoff=-1.45535) **(E)**, GSE50081 (cutoff=0.8180871) **(F)** cohorts.

**(G-L)** Kaplan-Meier curves of overall survival between the low and high risk groups in different subgroups of TCGA-LUAD, including TNM stage **(G, H)**, age **(I, J)** and gender **(K, L)**.

**
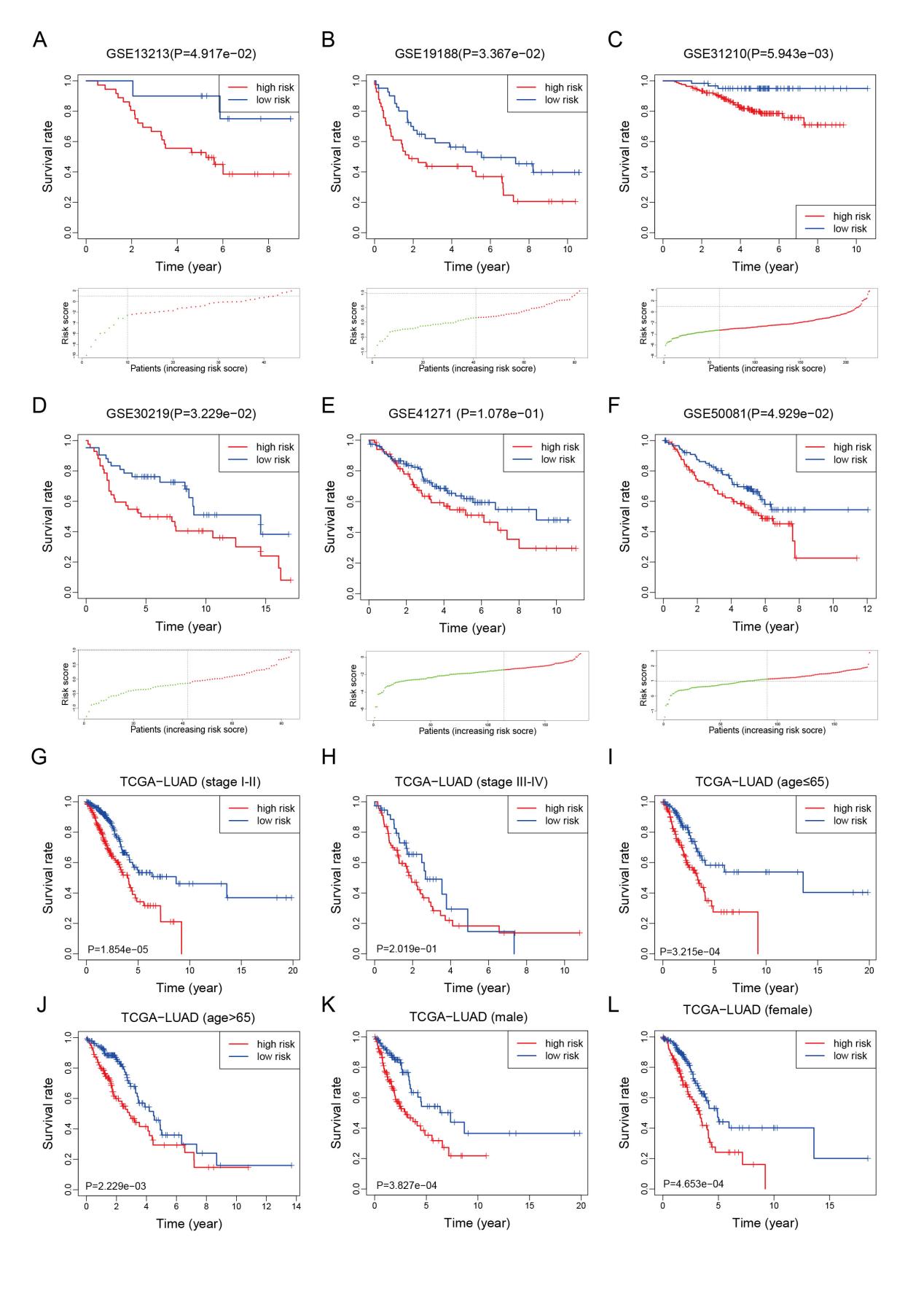
**

**Figure 5 Survival analysis of the four signature genes and validation of the signature in CHCAMS cohort.**

1. **D**) Kaplan-Meier survival analysis of the four signature genes in TCGA-LUAD. (**E-H**) Kaplan-Meier survival analysis of the four signature genes in CHCAMS cohort.
2. Kaplan-Meier survival analysis of the signature in CHCAMS cohort.

(**J**) Representative images of the immunohistochemistry results in CHCAMS cohort.

TCGA-LUAD, lung adenocarcinoma cohort from The Cancer Genome Atlas; CHCAMS, Cancer Hospital, Chinese Academy of Medical Sciences.

**
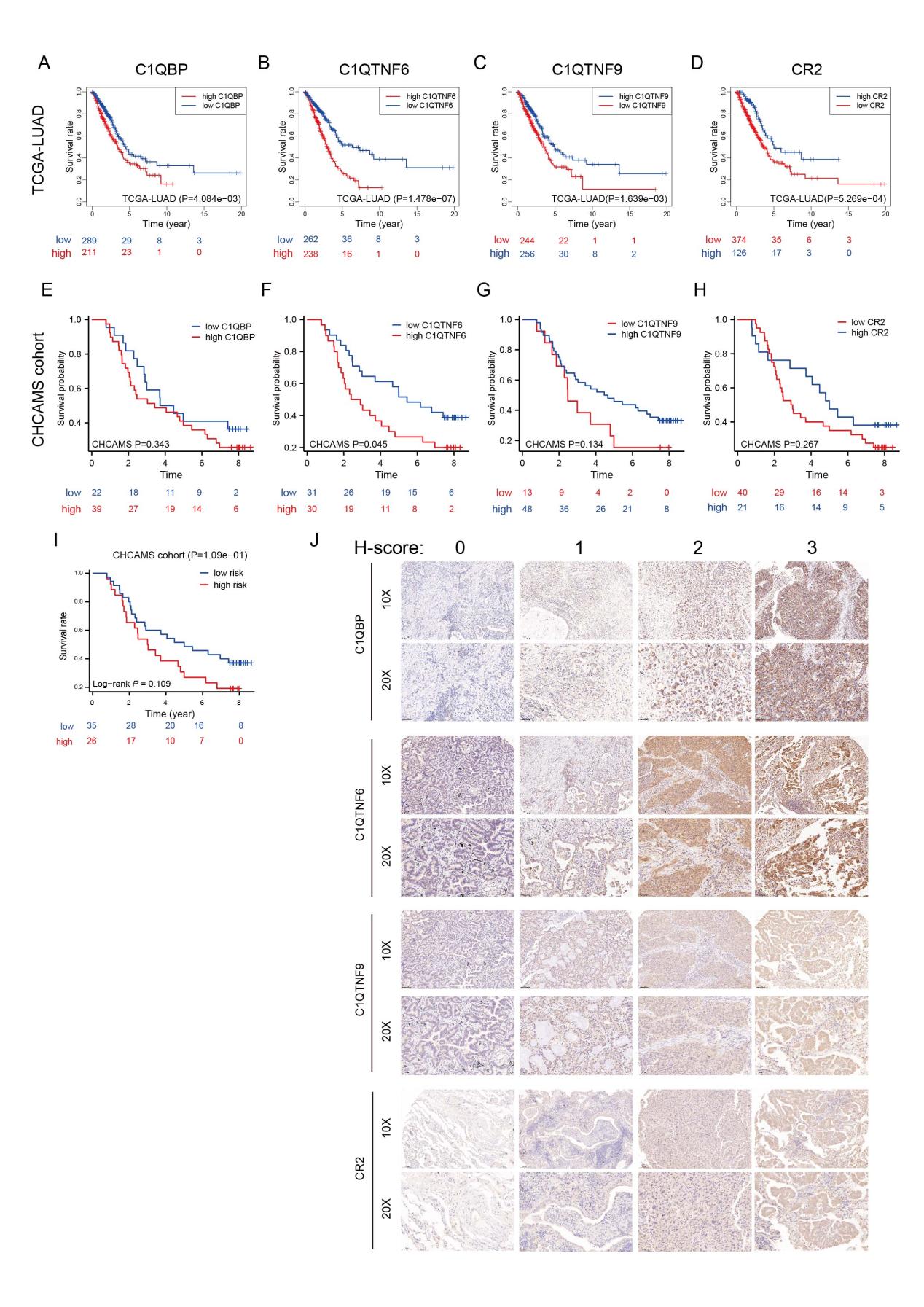
**

**Figure 6 Infiltrated immune cells assessment and biological pathways enrichment analysis.**

**(A)** Assessing the abundance of infiltrated immune cells between the low and high risk groups. ^***^*P* < 0.001.

**(B-E)** Gene set enrichment analysis of GO terms and KEGG pathways positively **(B, D)** or negatively **(C, E)** correlated with risk scores.


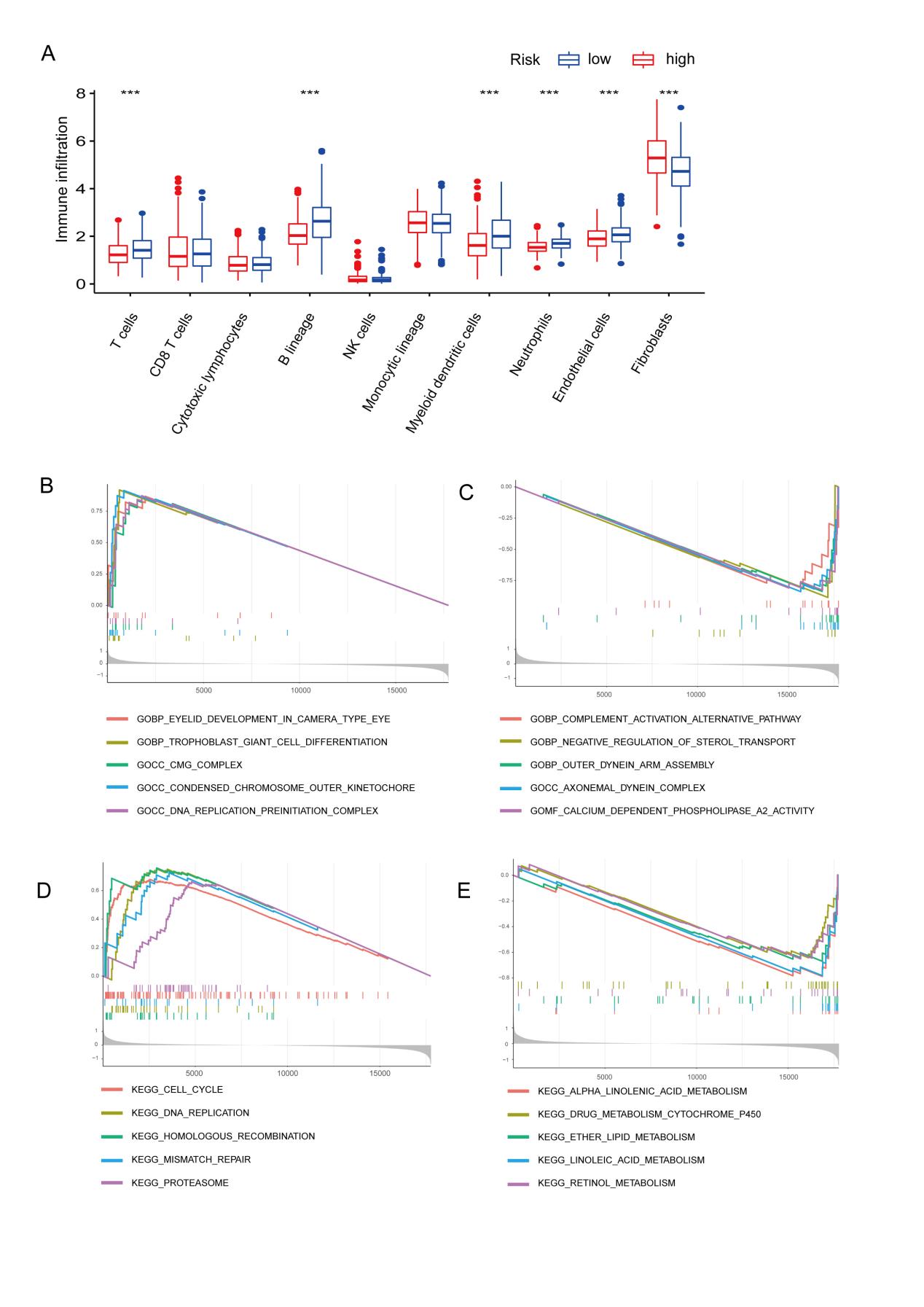


**Figure S1** Clustering based on the expression profile of the complement-related genes. (**A**) Heatmap of NMF clustering for complement-related genes in TCGA-LUAD cohort with cluster numbers from 2–7. (**B**) The relationships between cophenetic, dispersion, evar, residuals, rss, silhouette, sparseness coefficients and the numbers of clusters. The horizontal axis stands for k.

**
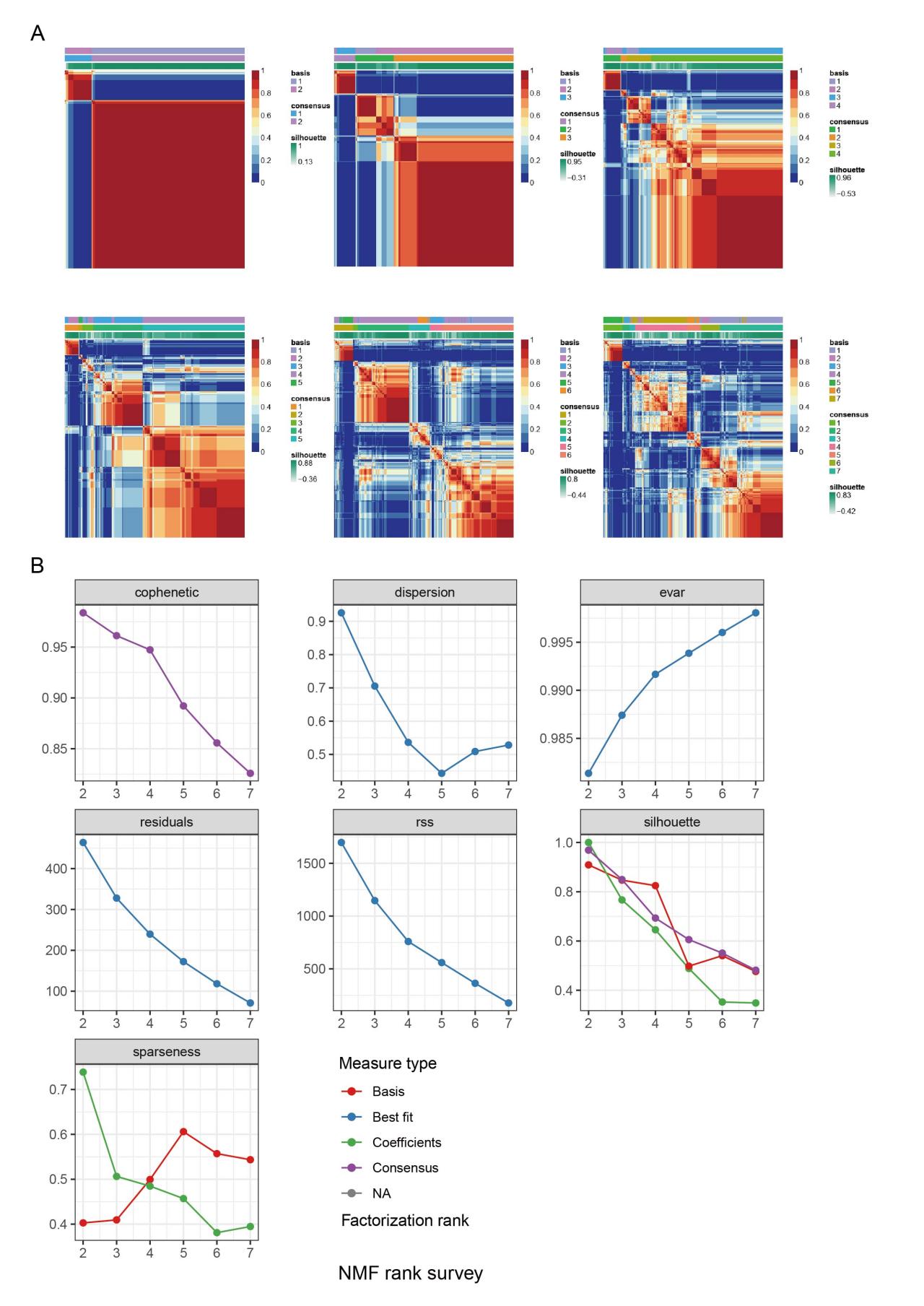
**

**Figure S2** Construction of the gene signature. (**A**) The selection of lambda in the LASSO model by 1000-fold cross-validation based on minimum criteria for overall survival. (**B**) LASSO coefficient profiles of the 7 complement related genes. (C) Forrest plot showing the 4 genes in the final model generated by the TCGA training cohort.
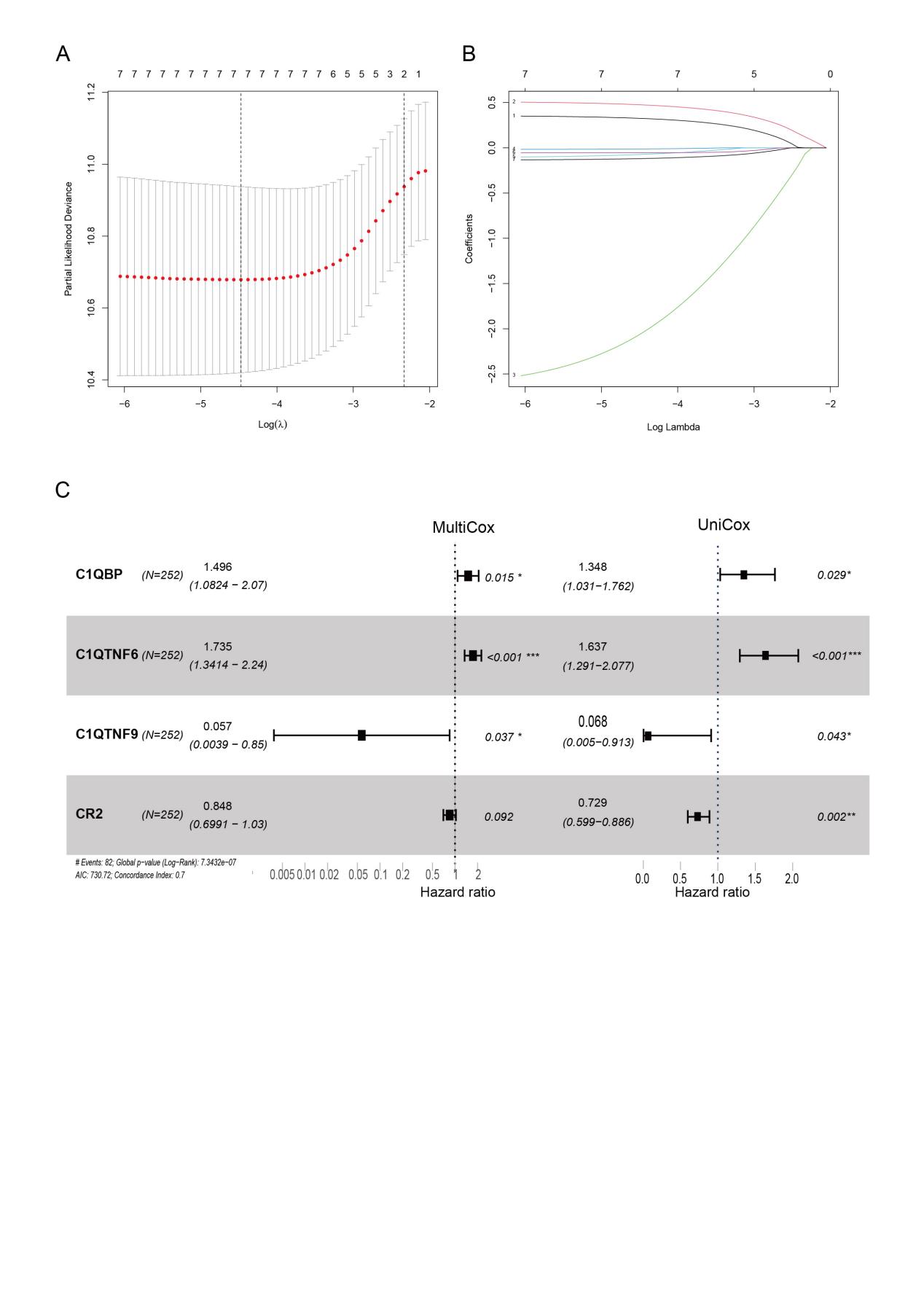


**Figure S3** (**A**) Correlations between the complement related genes in TCGA-LUAD. (**B**) Heatmap showing the expression profiles of the complement related genes in two risk groups in TCGA-LUAD.


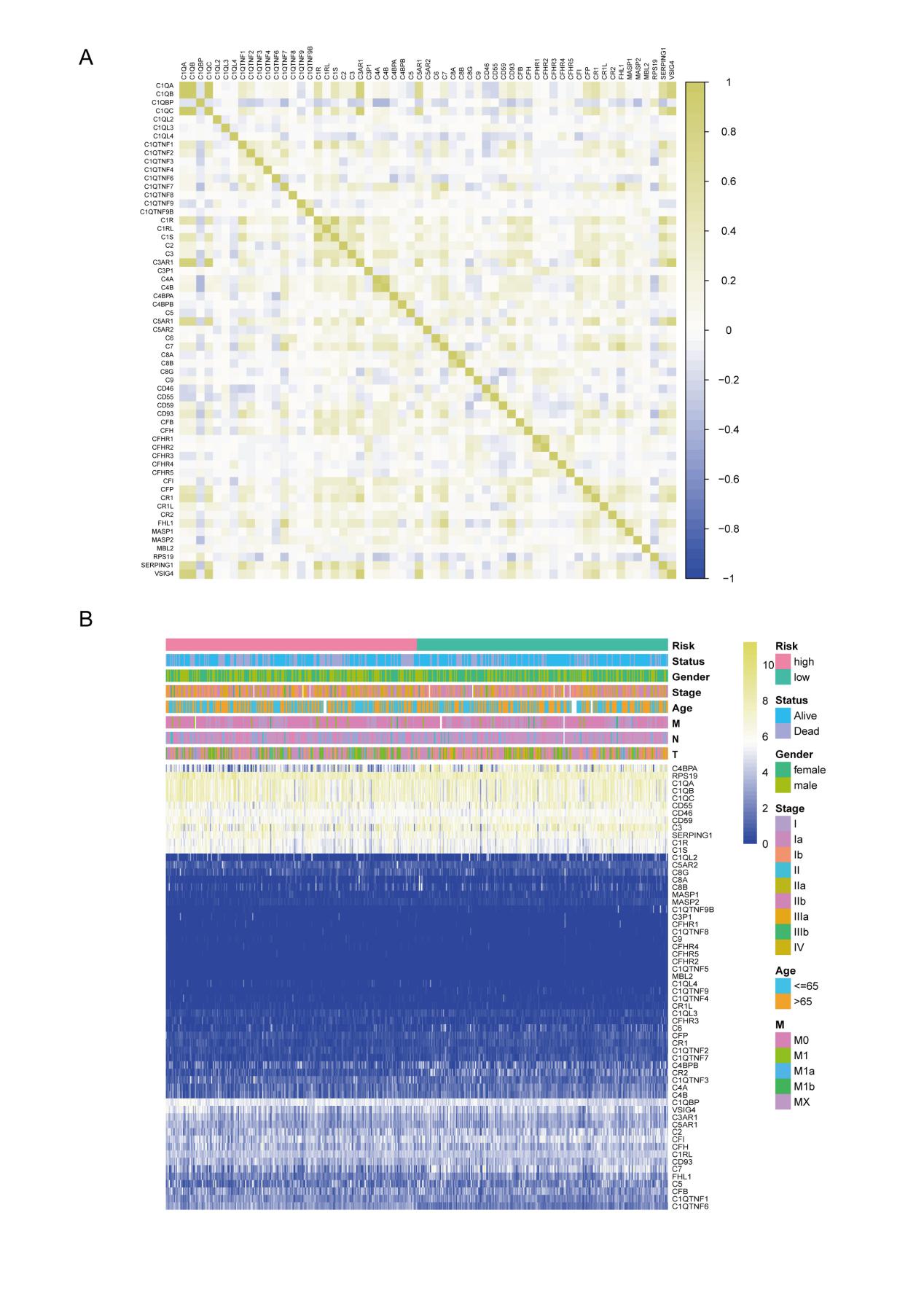


**Figure S4** Receiver operator characteristic curves of the complement-related gene signature in different datasets (**A-G**) and distribution of risk scores in different stages in TCGA-LUAD (**H-I**). (**J**) Comparison of immune infiltration between stage I-II and stage III-IV patients in TCGA-LUAD.

**
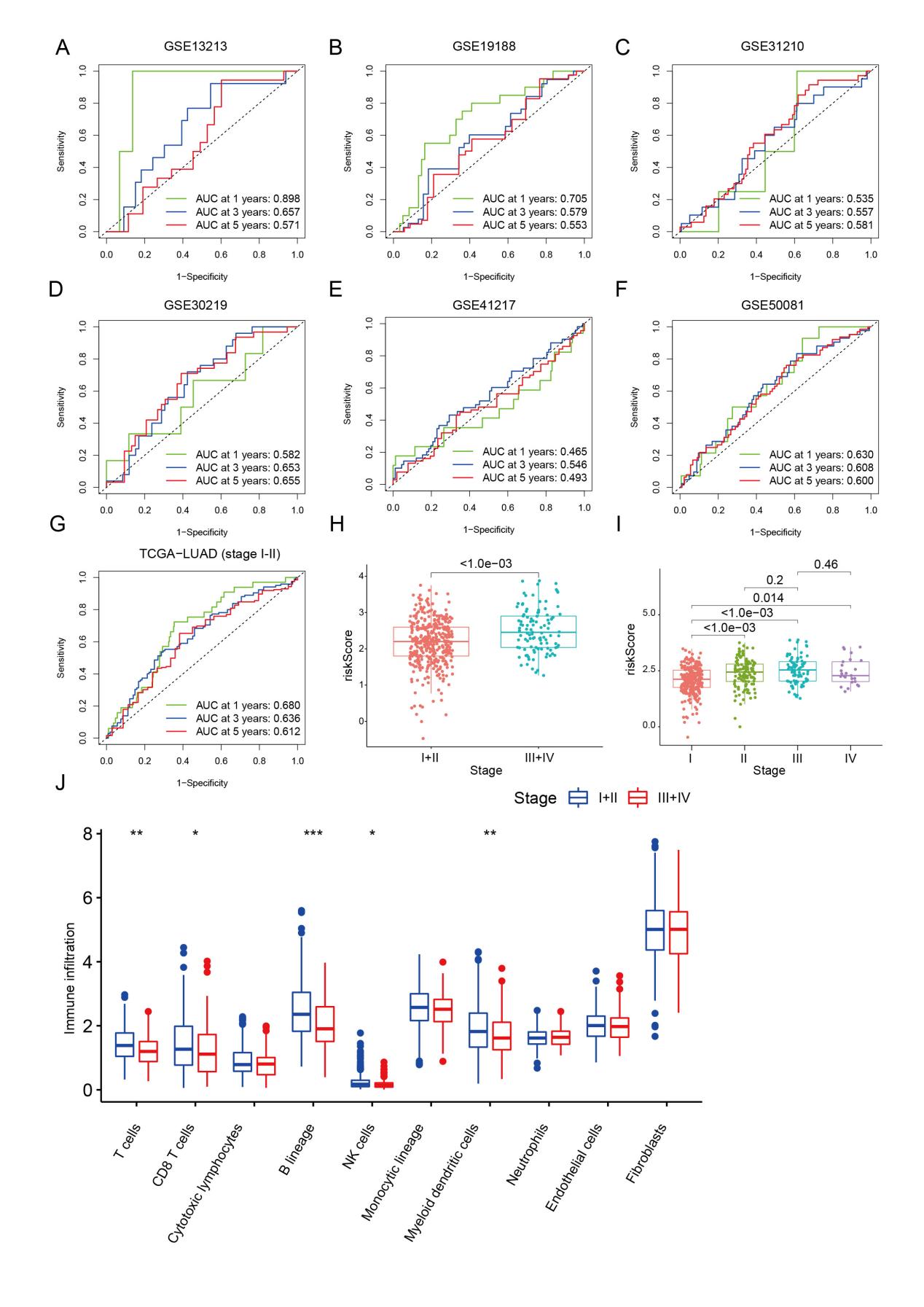
**

**Figure S5** The top 10 significant GO terms (**A**) and KEGG pathways (**B**) positively or negatively correlated to risk scores identified by gene set variation analysis. (**C**) Comparison of Estimation of STromal and Immune Cells in MAlignant Tumours using Expression Data scores between low and high risk groups. (**D**) Comparison of expressions of several prominent immune checkpoint genes between low and high risk groups.

**
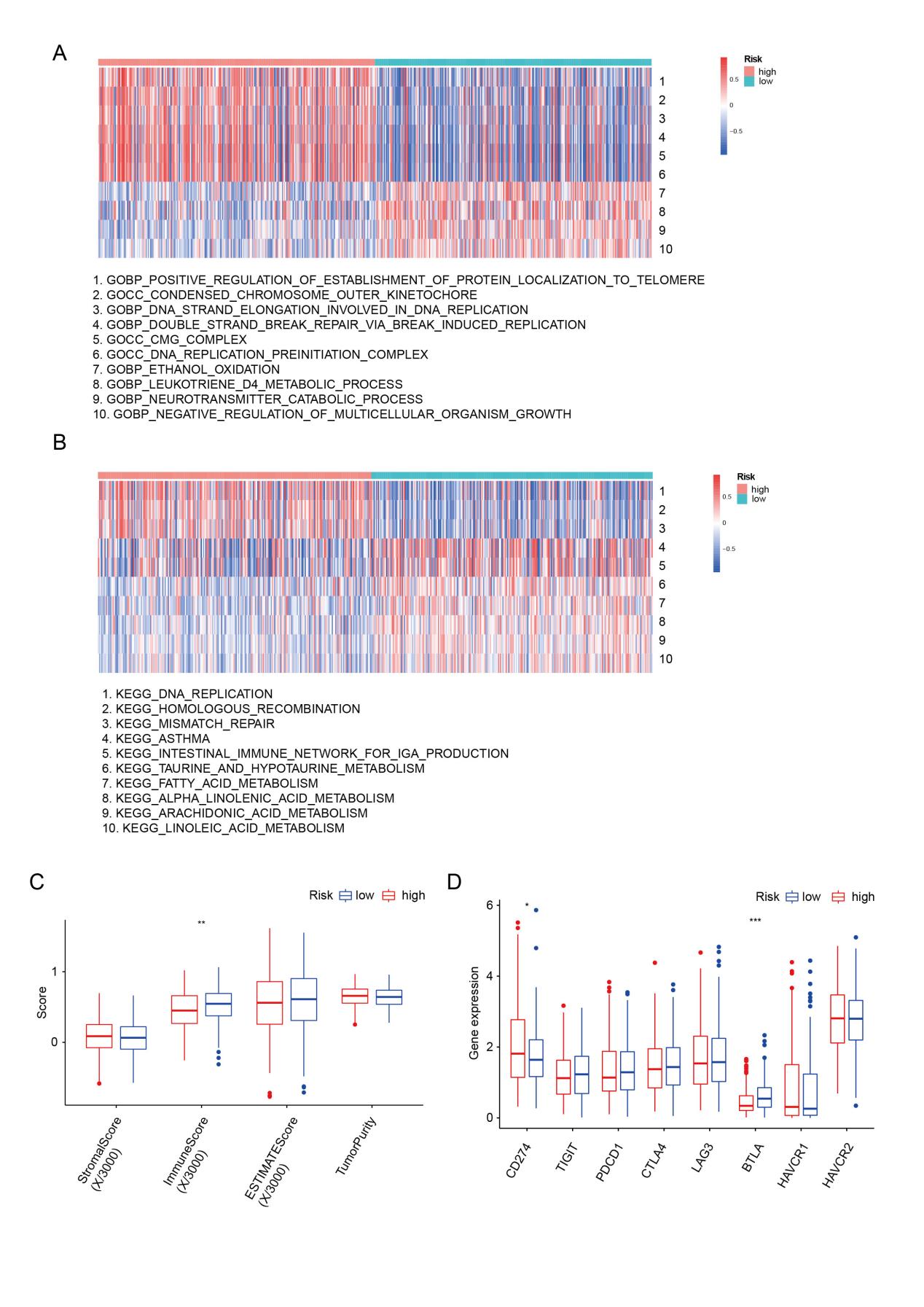
**
